# The prevalence of subthreshold psychiatric symptoms and associations with alcohol and substance use disorders: from a nationally representative survey of 36,309 adults

**DOI:** 10.1186/s12888-022-03834-1

**Published:** 2022-04-15

**Authors:** Jeremy C. S. Johnson, Gerard J. Byrne, Anita M. Pelecanos

**Affiliations:** 1 The University of Queensland, Mental Health Centre, K Floor, Royal Brisbane & Women’s Hospital, Herston, QLD 4029 Australia; 2 Department of Psychiatry, Royal Brisbane & Women’s Hospital, Butterfield St, Herston, QLD 4029 Australia; 3 Department of Psychiatry, Older Persons’ Mental Health Service, Royal Brisbane & Women’s Hospital, Butterfield St, Herston, QLD 4029 Australia; 4 Statistics Unit, QIMR Berghofer Medical Research Institute, 300 Herston Rd, Herston, QLD 4006 Australia

**Keywords:** Alcohol use disorders, Drug use disorders, Subclinical, Substance use disorders, Subthreshold, Subsyndromal

## Abstract

**Background:**

Our aim was to describe a broad number of subthreshold psychiatric symptoms (SPS) in a nationally representative population and evaluate associations with substance use. SPS describe groups of symptoms with significant pathology, but that do not quite meet full psychiatric diagnostic criteria. They have been associated with significant impairment and cost.

**Methods:**

The National Epidemiologic Survey on Alcohol and Related Conditions-III was a multistage, weighted, cross-sectional survey completed in the United States in 2013 comprising 36,309 noninstitutionalized adults. We report lifetime prevalence rates of 14 SPS related to mood, anxiety, trauma, eating, and personality disorders. We then evaluate associations with lifetime alcohol use disorders (AUD) and all substance use disorders (SUD) using logistic regression and adjusted odds ratios. SPS and psychiatric diagnoses were mutually exclusive (could not co-occur).

**Results:**

Lifetime prevalence of having at least one of 14 SPS was 57% compared with 37% for the related psychiatric disorders. This was similar for males and females, in contrast to psychiatric disorders in which prevalence was 42% in females and 31% in males. Otherwise, overall SPS and disorders had similar prevalence patterns across sociodemographic characteristics. Subthreshold personality symptoms had the highest prevalence rates (schizotypal 21.3%, antisocial 18.3%, and borderline 17.6%), followed by posttraumatic stress (13.1%). Subthreshold bipolar and depression had lifetime prevalence rates of 2.7 and 8.5%, respectively. Prevalence rates of subthreshold anxiety symptoms ranged from 2.2% (agoraphobia) to 9.8% (specific phobia). Subthreshold eating disorder related symptoms had the lowest prevalence rates (anorexia 1.5% and bulimia 1.7%). Half (seven) of the SPS had significantly increased odds of lifetime AUD. This number increased to 12 for all SUD. Subthreshold antisocial personality symptoms had the highest odds of AUD (2.2; 95% CI 2.00–2.37) and SUD (3.5; 95% CI 3.22–3.81).

**Conclusions:**

We found high lifetime SPS prevalence rates and significant associations with AUD and SUD. To our knowledge, this is the first published study evaluating a broad number of SPS. This indicates possible opportunities for early intervention and prevention but requires additional research and development of infrastructure and guidelines to better understand and manage patients who experience SPS.

**Supplementary Information:**

The online version contains supplementary material available at 10.1186/s12888-022-03834-1.

## Background

Subthreshold psychiatric symptoms (SPS) are groups of symptoms with significant pathology that do not quite meet all criteria for a mental disorder based on common diagnostic manuals. Within the literature there are various names used for SPS including ‘subclinical’ and less frequently ‘subsyndromal’. They have been associated with close to half the functional disability [1] and economic cost [2] related to mental illness, and may be associated with the risk of developing treatment resistant psychiatric disorders. This is well accepted for psychosis and is referred to as the prodromal phase of schizophrenia. Similarly, subthreshold depression has been associated with major depression [3–6]. Furthermore, there are advocates for defining SPS as a diagnostic category, such as for PTSD [7], rather than considering them as subthreshold symptoms. Some findings suggest SPS have similar prevalence rates to their corresponding clinical diagnoses [6, 8], and share similar risk factors and comorbidities [3, 6, 9, 10]. Others have found higher prevalence rates, such as for subthreshold anxiety [10], trauma [11], personality [12, 13], and eating [14] related symptoms. SPS may be exacerbated by substance use disorders (SUD) and vice versa.

### SPS and substance use disorders

SUD in the The National Epidemiologic Survey on Alcohol and Related Conditions-III (NESARC-III) study consist of alcohol, tobacco, and drug use disorders, and are common comorbidities of other psychiatric disorders. Similarly, SPS have been associated with SUD. This has been demonstrated in different populations for various SPS including generalized anxiety [10, 15] and depression [16]. Additionally, there are dose-dependent associations, such that having more subthreshold symptoms increases the risk of developing SUD. For example, for borderline personality disorder comorbid SUD increased from 7 to 18% as symptom number increased from 1–2 to 3–4 symptoms, respectively [12]. There is also research suggesting gender differences, with males having higher rates of SUD in subthreshold obsessive compulsive disorder [17] and gambling disorder [18]. There have been some inconsistent findings, such as for social anxiety in which Miloyan and colleagues reported no association with AUD [19] and identified that this conflicted with previous findings. These differences, and limited relevant research, create an imperative for evaluating subthreshold symptoms in larger studies with diverse populations to elucidate associations between SPS and SUD.

### SPS and NESARC-III

The NESARC-III is a large cross-sectional survey administered in the United States (see Methods section for more detail). Gilbert and Marzell [20] assessed subthreshold alcohol use disorders, so-called “diagnostic orphans”, but there have been few published journal articles evaluating SPS using NESARC-III data. Goldstein et al. [21] evaluated subthreshold antisocial personality disorder in which conduct disorder was not present before the age 15. They found similar comorbidities, although the subthreshold form was more prevalent. The NESARC-III does not have SPS variants defined in its general dataset, however the survey allows for subthreshold categories to be determined from the raw data [22].

### Purpose and hypotheses

In this article we describe prevalence rates for 14 SPS and overall prevalence rate using the NESARC-III sample, and evaluate associations with AUD and SUD. Our purpose is to report data on a broad number of SPS from a single population, which, to our knowledge, has not been previously published in the literature. We hypothesize that SPS will have high prevalence rates and will be positively associated with AUD and SUD, relative to the asymptomatic population. Additionally, we provide prevalence rates for the related psychiatric disorders for comparative purposes.

## Methods

### Ethics & NESARC-III data

Ethical approval was granted by the University of Queensland Human Research Ethics Committee, and the dataset was obtained from the National Institute on Alcohol Abuse and Alcoholism (NIAAA) under the Data Use Agreement. NESARC-III was conducted by the NIAAA from 2012 to 2013. Multistage probability sampling resulted in a response rate of 60.1% and a final sample size of 36,309 non-institutionalized civilian adults from all fifty states of the United States. Additional sampling and methodological information has been published in detail elsewhere [23–25] and is available from the dedicated section of the NIAAA website [26].

### Survey format

The survey tool used was the Alcohol Use Disorder and Associated Disabilities Interview Schedule (AUDADIS-5) [27–29]. The AUDADIS-5 includes sections on demographics, detailed drug and alcohol information, and family and psychiatric histories intended to be consistent with the Diagnostic and Statistical Manual of Mental Disorders, 5th Edition (DSM-5). For diagnostic sections, screening questions were asked based on specific DSM-5 criteria (e.g. dysphoria and anhedonia for major depression). If insufficient criteria were met, then the rest of the section was skipped. Similarly, the questionnaires allowed for only parts of sections to be skipped if certain criteria were not met. This survey format guided the process of developing subthreshold categories.

### Dataset

Within the dataset provided by NIAAA, in addition to the raw data, there were pre-coded demographic and diagnostic variables. This included 14 pre-coded psychiatric disorders: bipolar I disorder, major depressive disorder, panic disorder, agoraphobia, specific phobia, social phobia, generalized anxiety disorder, posttraumatic stress disorder, anorexia nervosa, bulimia nervosa, binge eating disorder, and three personality disorders (schizotypal, borderline, antisocial). Substance use disorders were also pre-coded: alcohol use disorder, tobacco use disorder, and 10 drug use disorders [26, 27].

### Subthreshold variables—data processing

Subthreshold variables were coded using the relevant AUDADIS-5 sections. Definitions of SPS were guided by a review of the subthreshold literature, although due to the broad nature of this study, and variable SPS definitions in the literature, there were no direct comparisons made. The aim was to identify groups of symptoms that met most, but not all, of the DSM-5 criteria that may have significant pathology if a patient presented with those symptoms. Fourteen SPS (Table 1) were evaluated, related to the 14 psychiatric disorders listed above. Initially, multiple subtypes for each SPS were determined, and then combined into a single subthreshold variable, outlined in Table 1. For example, for major depressive disorder there were two main subthreshold subtypes: 1) meeting fewer than five symptoms (3 or 4 symptoms) from criterion A, but meeting all other DSM-5 criteria; 2) not reporting significant distress while meeting all other criteria. In both subtypes there had been a two-week period of daily dysphoria and/or anhedonia. The dysphoria and anhedonia could be self-reported or based on what others observed. For example, a question in the AUDADIS-5 enquiring about other’s observations of dysphoria asked: “have you ever had a time when other people noticed that you were SO sad, hopeless, depressed, or down that you weren’t your normal self or that they were concerned about you nearly every day for at least 2 weeks?” The various subtypes are similar to those seen within psychiatric disorders for which there is heterogeneity due to the format of the DSM-5. Due to the survey structure, there could be some subthreshold subtypes that were missed. Importantly, SPS and psychiatric disorders were mutually exclusive. For example, if they met criteria for major depressive disorder, then they did not meet the criteria for subthreshold depression.

**Table 1:** Subthreshold Psychiatric Symptoms (SPS) Subtypes—Based on DSM-5 Criteria

SPS	Criteria Not Met	*n*	Description
Bipolar	A	399	Hypomania except less than 4 days duration, other criteria met
	B	247	Excited/elated, < 3 criterion B symptoms, other criteria met
	B	243	Irritable, < 4 criterion B symptoms, other criteria met
	C	43	Denying severe impairment, other criteria met
	All subtypes	922	*n* = 10 excluded due to substance and medical condition induced
Depression	A	2379	< 5 criterion A symptoms, other criteria met
	B	816	Denying severe impairment or distress, other criteria met
	All subtypes	3115	*n* = 80 excluded due to substance induced or other medical conditions
Panic	A	396	< 4 criterion A symptoms, multiple panic attacks, other criteria met
	B	996	Other criteria met
	All subtypes	1347	*n* = 45 excluded due to substance or medical condition induced
Generalized Anxiety	B or D	455	Not difficult to control worry OR denying distress, other criteria met
	C	1510	< 3 criterion C symptoms, other criteria met
	All subtypes	1904	*n* = 61 excluded due to substance or medical condition induced
Specific Phobia	B	177	For all subtypes only the single criterion was not met
	C	85	
	D	1196	
	E	734	
	F	1458	
	All subtypes	3587	*n* = 63 excluded due to substance or medical condition induced
Social Anxiety	B	115	For all subtypes only the single criterion was not met
	C	361	
	D	81	
	E	386	
	F	355	
	G	322	
	All subtypes	1554	*n* = 66 excluded due to substance or medical condition induced
Agoraphobia	B	129	For all subtypes only the single criterion was not met
	C	135	
	D	27	
	E	207	
	F	280	
	G	69	
	All subtypes	781	*n* = 66 excluded due to substance or medical condition induced
Posttraumatic Stress	C	792	All but criteria F and G met
	D	326	All but criteria F and G met
	E	1973	All but criteria F and G met
	F	1626	All but criterion G met
	G	114	All other criteria met
	All subtypes	4831	
Anorexia	A	165	All other criteria met
	B	53	All other criteria met
	C	46	All other criteria met
	A or B or C	238	Low weight with significant distress and dysfunction
	All subtypes	502	
Bulimia	B or C	19	Recurrent binging & purging, not fully meeting criteria B or C
	D	18	Recurrent binging & purging & distress, denying weight / shape focus
	B, C, or D	595	Recurrent binging & purging, not meeting full criteria B, C, and/or D
	All subtypes	632	
Binge eating	A2	1018	Significant binging over 3 months although not always lack of control
	B	19	< 3 criteria B symptoms, other criteria met
	C	164	All other criteria met
	B and C	113	Significant binging with loss of control, not fully meeting criteria B and C
	All subtypes	1314	
Schizotypal Personality	A	5273	3 or 4 criterion A symptoms with distress or dysfunction
	C	2783	5 to 9 criterion A met, denying significant distress or dysfunction
	All subtypes	8056	
Borderline Personality	A	5402	3 or 4 criterion A symptoms with distress or dysfunction
	C	1205	5 to 9 criterion A met, denying significant distress or dysfunction
	All subtypes	6607	
Antisocial Personality	A	7337	3 or 4 criterion A symptoms with distress or dysfunction
	All subtypes	6683	*n* = 654 excluded due to substance and medical condition induced

### Statistical analysis

Statistical analysis was completed using Stata/SE 15 [30]. The NESARC-III sampling design used weighted proportions with standard error (SE) computed overall by demographic variables for presence of lifetime SPS and disorders. Lifetime rather than 12-month rates were used for this study since they were available for all disorders. Adjusted odds ratios (AOR) and 95% confidence intervals (CI) were computed using logistic regression models for the association between demographic variables and the presence or absence of each SPS. Demographic AOR were adjusted for all other demographics, consistent with previous studies that evaluated the NESARC-III data [23–25]. Similarly, reference groups were chosen to be consistent with those in the published literature. AOR with 95% CI for DSM-5 lifetime AUD and SUD were computed for each SPS and disorder relative to asymptomatic individuals while controlling for all demographics and all other disorders [23–25]. Statistical significance was defined at *p* < 0.05. For statistical completeness and comparative purposes we have also provided supplementary data of multinomial logistic regression to compute relative risk ratios of having each lifetime SPS or its related disorder relative to the asymptomatic population, for each demographic, adjusted for all other demographics. It is pertinent to note that Stata defines them as relative risk ratios, rather than AOR, as they are ratios of relative risks (whereas some statistical software terms them AOR).

## Results

Overall, there was a 57% (0.65%) lifetime prevalence (SE) of having at least one of the 14 SPS, compared with 37% (0.52%) prevalence of having at least one of the related psychiatric disorders (Table 2). For SPS, the prevalence was similar for females 59% (0.69%) and males 56% (0.76%), whereas for the psychiatric disorders it was 42% (0.62%) for females and 31% (0.63%) for males. Otherwise, overall SPS and disorders had similar prevalence patterns across sociodemographic characteristics.

**Table 2:** Overall Lifetime Prevalence of SPS and Psychiatric Disorders by Sociodemographic Characteristics

Demographic	Lifetime prevalence % (SE)	
	Any SPS	Any Disorder
Total	57.4 (0.65)	36.8 (0.52)
Sex		
Male	56.0 (0.76)	30.8 (0.63)
Female	58.6 (0.69)	42.4 (0.62)
Race / Ethnicity		
White	58.7 (0.75)	39.8 (0.63)
Black	61.1 (1.46)	32.9 (0.96)
Native American	72.9 (2.36)	53.0 (2.86)
Asian or Pacific Islander	44.7 (1.53)	21.9 (1.24)
Hispanic	51.5 (0.89)	30.7 (0.81)
Age		
18–29	61.1 (1.06)	38.4 (0.80)
30–44	58.0 (0.85)	38.4 (0.75)
45–64	57.7 (0.83)	39.0 (0.72)
≥ 65	51.0 (0.92)	28.4 (0.70)
Marital Status		
Married or cohabiting	53.8 (0.65)	33.8 (0.53)
Separated, widowed or divorced	63.3 (0.87)	43.6 (0.82)
Never married	61.3 (1.09)	38.8 (0.80)
Education		
Less than high school	56.4 (1.07)	35.9 (1.00)
High school	59.4 (0.95)	36.9 (0.77)
Some college or higher	56.7 (0.64)	37.0 (0.56)
Family Income		
0–19,999	62.7 (0.90)	42.5 (0.83)
20,000–34,999	59.6 (0.94)	38.5 (0.71)
35,000–69,999	57.7 (0.78)	35.6 (0.71)
≥ 70,000	51.8 (0.86)	32.8 (0.71)
Urbanicity		
Urban	57.0 (0.66)	36.4 (0.55)
Rural	58.8 (1.42)	38.3 (1.24)
Region		
Northeast	57.5 (1.77)	37.7 (1.03)
Midwest	58.0 (1.31)	37.2 (1.11)
South	57.1 (1.13)	35.9 (0.95)
West	57.1 (0.98)	37.3 (0.87)

Abbreviations: *SPS* subthreshold psychiattric symptoms, *SE* standard error

### Prevalence rates for 14 SPS

**Fig. 1:**
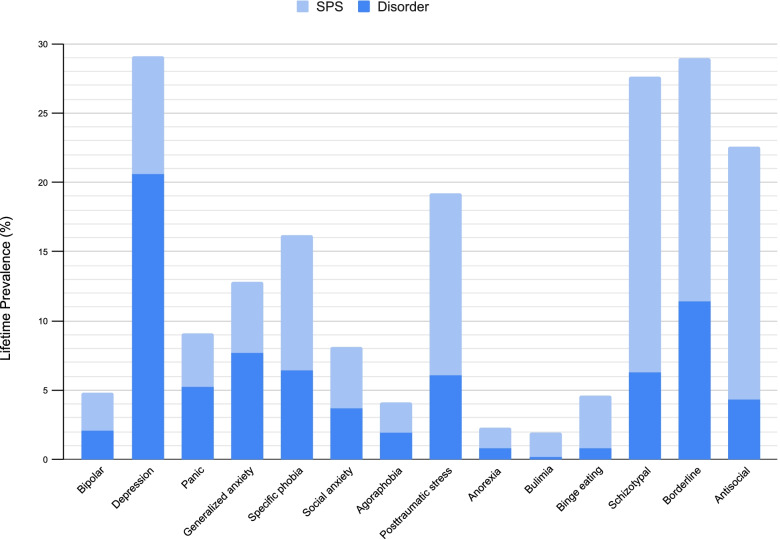
Lifetime Prevalence (%) of Subthreshold Psychiatric Symptoms (SPS) and Disorders

Figure 1 shows lifetime prevalence estimates for the 14 SPS (light shade) compared with the corresponding disorders (dark shade). Most mood and anxiety SPS had similar lifetime prevalence rates to their corresponding disorders, whereas trauma, eating, and personality SPS had higher lifetime prevalence rates than their corresponding disorders. The following lifetime prevalence rates are for SPS and disorder, respectively: bipolar 2.7% vs. 2.1%, depression 8.5% vs. 20.6%, panic 3.9% vs. 5.2%, generalized anxiety 5.1% vs. 7.7%, specific phobia 9.8% vs. 6.4%, social anxiety 4.4% vs. 3.7%, agoraphobia 2.2% vs. 1.9%, posttraumatic stress 13.1% vs. 6.1%, anorexia 1.5% vs. 0.8%, bulimia 1.7% vs. 0.2%, binge eating 3.8% vs. 0.8%, schizotypal 21.3% vs. 6.3%, borderline 17.6% vs. 11.4%, antisocial 18.3% vs. 4.3%. Lifetime prevalence estimates and SE for individual SPS by sociodemographic characteristics can be found in Supplement 1.

### Demographics associations

Table 3 shows the AOR (95% CI) for lifetime SPS by sociodemographic characteristics. Males had significantly higher odds of lifetime bipolar, binge eating, schizotypal and antisocial SPS, but lower odds of depression, panic, specific phobia, social anxiety, agoraphobia, posttraumatic stress, and anorexia SPS, compared to females. Asian ethnicity tended to have a lower odds of lifetime SPS, except for anorexia. Age groups less than 65 had significantly higher odds ratios for most SPS except for depression and generalized anxiety. Those not married or cohabiting had higher odds ratios for personality SPS. Respondents with family income brackets less than 70,000 had significantly higher odds of SPS, except for depression, anorexia, and bulimia. There were no clear trends for education, urbanicity, and region. Supplement 2 shows the relative risk ratios for lifetime SPS and disorders, relative to the asymptomatic population, for comparative and statistical purposes.

**Table 3:** Adjusted Odds Ratios (AOR) for Lifetime Subthreshold Psychiatric Symptoms (SPS) by Sociodemographic Characteristics

Demographic	SPS—AOR (95% CI)													
	bp	mdd	panic	gad	spp	sad	agor	ptsd	an	bn	bed	spd	bpd	aspd
Sex														
Male	**1.3**	**0.8**	**0.7**	0.9	**0.6**	**0.7**	**0.6**	**0.7**	**0.2**	0.9	**2.5**	**1.1**	1.1	**1.7**
	(1.10–1.54)	(0.74–0.88)	(0.58–0.77)	(0.83–1.07)	(0.52–0.67)	(0.64–0.85)	(0.48–0.66)	(0.61–0.74)	(0.14–0.28)	(0.76–1.10)	(2.14–2.86)	(1.04–1.18)	(0.99–1.13)	(1.54–1.78)
Female	REF	REF	REF	REF	REF	REF	REF	REF	REF	REF	REF	REF	REF	REF
Race / Ethnicity														
White	REF	REF	REF	REF	REF	REF	REF	REF	REF	REF	REF	REF	REF	REF
Black	1.0	1.0	**0.4**	**1.3**	1.0	**0.6**	**0.5**	0.9	**0.4**	0.9	1.0	**1.3**	1.1	**0.9**
	(0.83–1.27)	(0.92–1.19)	(0.29–0.44)	(1.13–1.58)	(0.84–1.09)	(0.45–0.70)	(0.39–0.69)	(0.82–1.02)	(0.22–0.58)	(0.69–1.30)	(0.85–1.28)	(1.15–1.42)	(1.00–1.25)	(0.78–1.01)
Native American	**2.0**	1.3	0.8	1.1	1.2	1.2	1.1	**1.4**	0.5	0.9	**1.8**	**1.5**	1.1	1.1
	(1.11–3.45)	(0.94–1.87)	(0.47–1.32)	(0.66–1.80)	(0.88–1.54)	(0.72–1.98)	(0.61–2.10)	(1.08–1.93)	(0.15–1.8)	(0.45–1.99)	(1.08–2.92)	(1.11–1.95)	(0.87–1.47)	(0.83–1.48)
Asian or Pacific Islander	0.8	0.9	**0.4**	0.9	**0.7**	**0.5**	**0.3**	**0.6**	1.2	**0.5**	**0.6**	**0.8**	**0.8**	**0.3**
	(0.57–1.19)	(0.69–1.07)	(0.30–0.61)	(0.66–1.15)	(0.58–0.85)	(0.36–0.82)	(0.18–0.62)	(0.53–0.75)	(0.76–1.99)	(0.28–0.89)	(0.41–0.76)	(0.68–0.99)	(0.64–0.91)	(0.24–0.36)
Hispanic	0.9	1.0	**0.5**	1.0	**0.8**	**0.5**	**0.6**	**0.7**	0.8	0.9	0.9	1.0	**0.8**	**0.5**
	(0.72–1.15)	(0.89–1.18)	(0.42–0.67)	(0.86–1.26)	(0.67–0.88)	(0.43–0.67)	(0.46–0.77)	(0.65–0.82)	(0.52–1.13)	(0.65–1.16)	(0.75–1.10)	(0.87–1.06)	(0.72–0.89)	(0.49–0.61)
Age														
18–29	**2.2**	**0.7**	**1.4**	**0.6**	**1.7**	**1.8**	**1.5**	**1.4**	**2.0**	**2.2**	**2.6**	**1.2**	**1.5**	**2.7**
	(1.64–2.99)	(0.61–0.85)	(1.09–1.88)	(0.46–0.71)	(1.40–2.03)	(1.39–2.24)	(1.04–2.04)	(1.17–1.62)	(1.05–3.86)	(1.46–3.25)	(1.97–3.32)	(1.07–1.40)	(1.33–1.80)	(2.33–3.02)
30–44	**2.1**	**0.8**	**1.5**	**0.6**	**1.5**	**1.6**	**1.5**	**1.6**	1.5	**2.1**	**2.0**	**1.1**	**1.4**	**2.8**
	(1.56–2.96)	(0.71–0.94)	(1.18–1.84)	(0.52–0.74)	(1.29–1.80)	(1.32–2.02)	(1.11–2.12)	(1.40–1.86)	(0.85–2.75)	(1.37–3.30)	(1.52–2.57)	(1.02–1.29)	(1.27–1.61)	(2.42–3.16)
45–64	**1.8**	1.0	**1.7**	**0.8**	**1.4**	**1.4**	**1.4**	**1.5**	1.2	**2.2**	**1.7**	**1.1**	**1.3**	**2.1**
	(1.32–2.37)	(0.84–1.08)	(1.34–2.05)	(0.67–0.91)	(1.21–1.62)	(1.19–1.73)	(1.04–1.80)	(1.34–1.74)	(0.66–2.22)	(1.58–3.16)	(1.28–2.16)	(1.05–1.25)	(1.12–1.40)	(1.82–2.35)
≥ 65	REF	REF	REF	REF	REF	REF	REF	REF	REF	REF	REF	REF	REF	REF
Marital Status														
Married or cohabiting	REF	REF	REF	REF	REF	REF	REF	REF	REF	REF	REF	REF	REF	REF
Separated, widowed or divorced	1.1	**1.3**	1.1	1.1	1.0	1.2	1.0	**1.4**	1.1	1.0	1.1	**1.3**	**1.3**	**1.4**
	(0.85–1.31)	(1.14–1.40)	(0.96–1.37)	(0.95–1.29)	(0.90–1.11)	(0.99–1.41)	(0.79–1.28)	(1.23–1.48)	(0.77–1.64)	(0.75–1.24)	(0.85–1.30)	(1.18–1.39)	(1.20–1.41)	(1.32–1.51)
Never married	1.2	1.1	0.9	1.1	0.9	1.1	1.2	1.0	**0.6**	1.3	**1.2**	**1.2**	**1.2**	1.1
	(0.97–1.42)	(0.96–1.25)	(0.76–1.18)	(0.94–1.28)	(0.77–1.00)	(0.94–1.29)	(0.87–1.51)	(0.89–1.12)	(0.39–0.96)	(0.97–1.65)	(1.04–1.44)	(1.07–1.28)	(1.08–1.32)	(1.00–1.21)
Education														
Less than high school	1.0	0.9	0.9	0.9	1.1	1.0	1.3	0.9	0.7	1.1	0.9	1.0	1.0	0.9
	(0.75–1.23)	(0.79–1.04)	(0.74–1.11)	(0.77–1.16)	(0.97–1.30)	(0.79–1.16)	(0.95–1.77)	(0.78–1.00)	(0.44–1.26)	(0.80–1.43)	(0.70–1.07)	(0.87–1.06)	(0.94–1.14)	(0.81–1.03)
High school	1.0	1.0	1.1	0.9	**1.1**	1.1	1.2	1.0	0.8	0.9	1.0	1.1	**1.1**	**1.1**
	(0.82–1.24)	(0.87–1.10)	(0.90–1.32)	(0.77–1.05)	(1.02–1.24)	(0.92–1.26)	(0.99–1.54)	(0.88–1.05)	(0.53–1.22)	(0.72–1.13)	(0.81–1.13)	(0.98–1.14)	(1.04–1.24)	(1.02–1.19)
Some college or higher	REF	REF	REF	REF	REF	REF	REF	REF	REF	REF	REF	REF	REF	REF
Family Income														
0–19,999	**1.5**	1.1	**1.6**	**1.3**	**1.2**	**1.4**	**1.8**	**1.4**	1.3	1.4	**1.3**	**1.5**	**1.3**	**1.6**
	(1.12–2.03)	(0.90–1.22)	(1.26–2.07)	(1.00–1.59)	(1.07–1.42)	(1.15–1.75)	(1.29–2.56)	(1.21–1.54)	(0.77–2.15)	(0.99–1.87)	(1.03–1.58)	(1.35–1.65)	(1.21–1.44)	(1.40–1.76)
20,000–34,999	**1.4**	0.9	1.3	**1.4**	**1.2**	1.3	**1.5**	**1.2**	1.2	1.0	1.1	**1.4**	**1.3**	**1.5**
	(1.11–1.85)	(0.82–1.06)	(1.00–1.67)	(1.11–1.68)	(1.08–1.44)	(0.98–1.62)	(1.02–2.08)	(1.06–1.34)	(0.71–1.91)	(0.72–1.43)	(0.88–1.38)	(1.24–1.53)	(1.14–1.40)	(1.35–1.66)
35,000–69,999	**1.4**	0.9	**1.3**	**1.2**	**1.2**	1.1	1.2	1.1	1.1	1.0	1.2	**1.3**	**1.3**	**1.3**
	(1.16–1.77)	(0.81–1.03)	(1.03–1.56)	(1.01–1.53)	(1.08–1.41)	(0.96–1.37)	(0.92–1.67)	(0.97–1.22)	(0.77–1.68)	(0.74–1.37)	(0.96–1.40)	(1.17–1.39)	(1.23–1.46)	(1.19–1.43)
≥ 70,000	REF	REF	REF	REF	REF	REF	REF	REF	REF	REF	REF	REF	REF	REF
Urbanicity														
Urban	0.9	**1.2**	1.1	1.0	0.9	0.9	1.1	1.0	1.0	1.1	1.1	0.9	1.0	1.0
	(0.71–1.13)	(1.03–1.30)	(0.93–1.33)	(0.85–1.21)	(0.75–1.05)	(0.77–1.13)	(0.89–1.40)	(0.88–1.09)	(0.62–1.54)	(0.79–1.48)	(0.83–1.37)	(0.85–1.06)	(0.90–1.15)	(0.89–1.16)
Rural	REF	REF	REF	REF	REF	REF	REF	REF	REF	REF	REF	REF	REF	REF
Region														
Northeast	1.1	1.1	1.1	1.1	1.1	**1.2**	1.3	0.9	0.8	0.9	1.0	1.0	0.9	0.9
	(0.84–1.56)	(0.88–1.35)	(0.94–1.37)	(0.82–1.48)	(0.94–1.27)	(1.00–1.44)	(0.96–1.65)	(0.79–1.07)	(0.48–1.41)	(0.74–1.16)	(0.76–1.24)	(0.87–1.10)	(0.81–1.06)	(0.72–1.07)
Midwest	0.8	**1.2**	0.9	0.9	**1.2**	1.1	1.0	0.9	0.7	0.9	0.9	0.9	1.0	**0.8**
	(0.61–1.12)	(1.01–1.32)	(0.75–1.10)	(0.69–1.11)	(1.05–1.36)	(0.90–1.34)	(0.78–1.28)	(0.75–1.02)	(0.42–1.13)	(0.67–1.21)	(0.73–1.20)	(0.79–1.04)	(0.86–1.14)	(0.65–0.90)
South	0.9	1.0	0.9	0.9	1.0	1.0	0.9	0.9	**0.5**	**0.8**	0.9	0.9	1.0	0.9
	(0.70–1.16)	(0.89–1.17)	(0.76–1.02)	(0.75–1.14)	(0.92–1.18)	(0.81–1.13)	(0.67–1.12)	(0.77–1.01)	(0.32–0.89)	(0.61–0.99)	(0.72–1.12)	(0.84–1.08)	(0.92–1.14)	(0.79–1.02)
	REF	REF	REF	REF	REF	REF	REF	REF	REF	REF	REF	REF	REF	REF

Abbreviations: *CI* confidence interval, *bp* bipolar, *mdd* depression, *gad* generalized anxiety, *spp* specific phobia, *sad*, social anxiety, *agor* agoraphobia, *ptsd* posttraumatic stress, *an* anorexia, *bn* bulimia, *bed* binge eating, *spd* schizotypal, *bpd* borderline, *aspd* antisocial

REF is the reference group for each demographic category (AOR = 1.0)

Significance at *p* < 0.05 in **bold.** AOR adjusted for all other demographics

### AUD and SUD associations

Table 4 shows AOR (95% CI) for lifetime AUD and SUD for the 14 SPS and related psychiatric disorders. Half (seven) of the SPS had significantly increased odds of lifetime AUD compared to the asymptomatic general population: depression, social anxiety, posttraumatic stress, binge eating, borderline, schizotypal and antisocial. This number increased to 12 for SUD (subthreshold agoraphobia and bulimia were not associated with increased odds of lifetime SUD). Subthreshold antisocial symptoms had the highest odds of lifetime AUD (2.2, 95% CI 2.00–2.37) and SUD (3.5, 95% CI 3.22–3.81).

**Table 4:** AOR of Lifetime AUD and SUD by Subthreshold and Psychiatric Disorder

Psychiatric Category	AOR (95%CI)			
	Lifetime AUD		Lifetime SUD	
	Subthreshold	Disorder	Subthreshold	Disorder
Bipolar	1.2 (0.94–1.40)	**2.1 (1.66–2.67)**	**1.4 (1.18–1.77)**	**3.0 (2.23–3.99)**
Depression	**1.1 (1.01–1.30)**	**1.4 (1.30–1.56)**	**1.2 (1.11–1.35)**	**1.7 (1.54–1.80)**
Panic	1.2 (0.96–1.39)	**1.2 (1.07–1.44)**	**1.4 (1.21–1.71)**	**1.8 (1.56–2.13)**
Agoraphobia	1.0 (0.81–1.28)	0.9 (0.65–1.14)	1.1 (0.87–1.36)	1.1 (0.84–1.48)
Specific phobia	1.1 (0.98–1.25)	**1.3 (1.16–1.51)**	**1.2 (1.12–1.39)**	**1.5 (1.37–1.75)**
Social anxiety	**1.3 (1.09–1.49)**	1.0 (0.82–1.19)	**1.3 (1.10–1.45)**	1.2 (0.99–1.37)
Generalized anxiety	1.1 (0.89–1.31)	**1.2 (1.02–1.37)**	**1.2 (1.03–1.32)**	**1.3 (1.17–1.47)**
Posttraumatic stress	**1.4 (1.26–1.50)**	**1.3 (1.12–1.54)**	**1.6 (1.51–1.78)**	**1.8 (1.53–2.02)**
Anorexia	1.0 (0.78–1.41)	**1.7 (1.26–2.21)**	**1.4 (1.05–1.81)**	1.4 (0.93–2.00)
Bulimia	1.0 (0.65–1.47)	1.8 (0.90–3.78)	1.2 (0.88–1.73)	1.4 (0.64–2.84)
Binge eating	**1.4 (1.14–1.64)**	1.3 (0.89–1.85)	**1.7 (1.40–1.97)**	1.3 (0.90–1.98)
Borderline personality	**1.8 (1.62–1.93)**	**2.4 (2.15–2.77)**	**2.2 (2.03–2.39)**	**3.7 (3.26–4.17)**
Schizotypal personality	**1.2 (1.14–1.33)**	1.1 (0.91–1.30)	**1.5 (1.37–1.65)**	1.2 (0.98–1.38)
Antisocial personality	**2.2 (2.00–2.37)**	**2.4 (2.02–2.79)**	**3.5 (3.22–3.81)**	**5.1 (4.18–6.15)**

Abbreviations: *AOR* adjusted odds ratios, *CI* confidence intervals, *AUD* alcohol use disorder, *SUD* substance use disorder

Significance at *p* < 0.05 in **bold**

AOR adjusted for all demographics, and all other psychiatric and substance use disorders

## Discussion

### Prevalence of SPS

We have reported data on 14 SPS for the NESARC-III sample. The lifetime prevalence of having at least one SPS was 57%, compared with 37% for the related disorders. This difference may seem like a necessary outcome due to SPS having less criteria to meet, however it is pertinent to reiterate that the subthreshold and psychiatric disorder categories were mutually exclusive in this study (i.e. if they met psychiatric diagnostic criteria, then they could not be categorised as having an SPS).

Higher prevalence rates of SPS compared to their related disorders were observed in trauma, eating, and personality categories. Whereas there were similar rates in the mood and anxiety categories, except for subthreshold depression that had less than half the lifetime prevalence (8.5%) of major depression (20.6%). This difference for depression is consistent with results in a general population study from the Netherlands [3]. Other studies, such as in older adults in the first wave of NESARC, have reported similar lifetime prevalence rates of ~ 14% [6] for both subthreshold and major depression. Similarly, a higher prevalence for subthreshold bipolar (4.6%) was reported in a study from Italy [31], compared to our finding (2.7%). They used the Florence Psychiatric Inventory, which grades symptoms from absent to severe, allowing for additional SPS to be identified.

The varying assessment formats modulate differences in prevalence rates reported. For example, the AUDADIS-5 depression section requires two weeks of dysphoria or anhedonia to be present. This is a requirement in some studies on subthreshold depression but not in others, which has been contemplated for decades [32] and assessed in systematic reviews [5, 33]. Other SPS criteria are also contentious, such as for subthreshold posttraumatic stress [7, 34, 35], personality [13], and generalized anxiety [10, 36]. One study found that self-reporting tools were more useful for evaluating subthreshold generalized anxiety [37]. Overall, this suggests that future research may benefit from standardized subthreshold assessment tools and SPS criteria.

The lower prevalence rates of SPS within the anxiety categories was likely modulated by how the subthreshold categories were formed. For anxiety categories it was possible to create several subtypes with all but one of the non-core criteria met. This provided a refined subthreshold category, but likely resulted in underestimation of prevalence rates. In the literature there are a variety of suggested criteria for subthreshold anxiety, some of which are defined more broadly than others. For example, there were multiple definitions of subthreshold generalized anxiety discussed in a systematic review of 18 studies [10], in which they concluded that there was double the prevalence compared to the full psychiatric disorder. Higher prevalence rates have also been reported for subthreshold panic [38] and social anxiety [39, 40].

Our finding of higher prevalence for subthreshold posttraumatic stress (13.1%) compared to the disorder (6.1%) is consistent with a 2016 meta-analysis [11] subthreshold finding of 12.6% among the highest quality studies. Similar to our study, previously published literature in a variety of populations reports higher prevalence rates for eating [41, 42], and personality [12, 13, 21] SPS compared with the disorders.

### Demographics associations

The novel finding that males had similar overall prevalence to females (of having any SPS) contrasts with the higher prevalence of psychiatric disorders found in females. This may account for some of the gender differences seen in population-based epidemiological studies [43, 44]. It may be that men are under-reporting their symptoms, or that higher substance use disorders in males [44] are moderating or masking some of the symptoms. The lower odds of SPS for the Asian ethnicity is consistent with findings from a study of a similar population in which psychiatric disorders were assessed and discussed in detail by Xu et al. [45]. Overall, they found that the Asian American/Pacific Islanders group had lower odds of having any past 12-month psychiatric disorder and SUD, compared with the non-Hispanic white group. Lower family income generally had higher odds of SPS, consistent with previous findings for psychiatric disorders [46] for which low socioeconomic status is a well-known risk factor. The comparison of prevalence rates across demographics for each SPS was not delineated herein due to the breadth of our study, although can be seen in Supplement 1.

### AUD and SUD associations

Overall, our findings of increased odds of AUD and SUD for various SPS are consistent with previous studies [12, 15, 16, 19, 39, 40, 47]. The 14 SPS had more associated comorbid alcohol and substance use relative to the asymptomatic general population, when adjusted for demographics and all other disorders. Twelve had a statistically significantly increased odds of SUD, four more than the disorders (binge eating, anorexia, social anxiety, and schizotypal personality). This finding is surprising, but the disorders had smaller prevalence rates with odds ratios approaching significance. Subthreshold antisocial, borderline, and posttraumatic stress had high odds of lifetime AUD and SUD, which is expected as the associated psychiatric disorders are known to have significant substance use comorbidity.

Subthreshold social anxiety and schizotypal personality were significantly associated with AUD and SUD (but not the disorders) suggesting that these SPS may be of interest when assessing for substance use. Consistent with these findings, a 15-year longitudinal study from Switzerland reported similar results for social anxiety [40]. They found that subthreshold social anxiety was associated with AUD and SUD, whereas social anxiety disorder, or having too few symptoms to be classed as SPS, were not. A study examining schizotypal personality symptoms reported increased odds of substance use based on schizotypy domain, in a non-psychiatric sample [48]. An evaluation of the NESARC-III schizotypal and social anxiety categories may identify specific symptoms that increase the risk of SUD.

Other NESARC-III publications have been unable to report on eating disorders due to being too rare [23–25]. This was not a concern for SPS and the results for disorders were included for comparative purposes, although should be interpreted with caution due to the small number of cases. The low rates of eating disorders (and larger CI) reflect the uncertainty in the estimates and make it more difficult to elucidate their relationship with AUD and SUD.

### Limitations, strengths, and future directions

Limitations and strengths specific to the NESARC-III have been considered elsewhere [23–29]. Nonetheless, it is pertinent to highlight that the cross-sectional survey data is limited to reporting associations. In addition, our results are limited to lifetime associations, which further limits assessment of temporal associations. However, twelve-month prevalence rates were not available for personality categories. The NESARC-III sample does not include institutionalised psychiatric patients, homeless individuals, nor deployed military personnel, thus we may be underestimating the SPS prevalence and must consider the generalisability of the results. Nonetheless, the sampling process and weighting methods provide a reasonable nationally representative sample. Conversely, overlapping diagnoses may inflate prevalence rates in this survey format, but also more broadly for psychiatric diagnoses in general. Logistic regression compensates for this overlap by adjusting for other disorders. Despite these survey limitations, the results provide an overview of a broad number of SPS and their associations with AUD and SUD in a single population, which has not been reported before.

A limitation in our study, and others evaluating SPS, is the formation of the subthreshold categories. There is a lack of consensus on how SPS should be defined. We identified multiple subthreshold subtypes, with the goal of creating meaningful SPS that did not meet DSM-5 criteria, but still had relevant symptoms. Evaluating SPS subtypes will be useful for delineating factors that may be associated with SUD [49]. Additionally, evaluating specific symptoms and dose-dependent relationships within each subtype could identify underlying mechanisms. A multi-dimensional approach, rather than categorical, may be useful for defining and understanding SPS. This has been eloquently recommended by various authors [6, 33], and may allow for earlier intervention and treatment, while minimizing semantics debates and fluctuating SPS definitions, which has been a longstanding burden to psychiatric nosology. This issue may be even more pertinent to SPS [50]. Despite potential benefits from a dimensional approach, we must be cautious of over-medicalising normal emotional and cognitive experiences [36]. Nonetheless, the boundaries between normal experiences, SPS, and psychiatric diagnoses are not definitive, and additional SPS research could help elucidate this nosological issue.

Those with lifetime psychiatric disorders may have had periods of time when they were classified as subthreshold, although this was not captured in our study. In current hierarchical classification systems, the disorder takes precedence over the subthreshold counterpart, but transitions can occur between them. Longitudinal and prospective studies will improve understanding of these transitions. Additionally, studies reporting statistical significance between SPS and psychiatric disorders will provide quantitative evidence for differences and similarities between these groups.

Although this may be one of the most broad studies to report on SPS, we only evaluated associations with substance use. Various studies have reported SPS associations with other psychiatric comorbidities [6, 12, 36, 38, 41, 47], functional disabilities [1, 36–38], psychosocial dysfunctions [12, 36] and physical comorbidities [3, 9], with SPS associations sometimes as large as those seen with their related disorders. This suggests that evaluating other comorbidities for subthreshold symptoms within the NESARC-III sample could be valuable.

## Conclusion

To our knowledge, this is the first study to evaluate a broad number of SPS and their associations with substance use within a single population. SPS have high lifetime prevalence rates and are significantly associated with AUD and SUD. This identifies potential opportunities for early intervention, and prevention of treatment resistant psychiatric disorders, but requires health workers to be armed with knowledge and treatment modalities. For this to occur, additional SPS research is needed, with support from governing bodies and reconsideration of health policies, to allocate new funding and consider relevant infrastructure and guidelines to better understand and support people experiencing SPS.

## Supplementary Information

**Additional file 1:** **Supplement 1.** Lifetime Prevalence for Subthreshold Psychiatric Symptoms (SPS) by sociodemographic characteristic.

**Additional file 2: Supplement 2.** Relative Risk Ratios (RRR) for Lifetime Subthreshold Psychiatric Symptoms (SPS) and Disorder by sociodemographic characteristics.

## Data Availability

The datasets analysed during the current study are available from NIAAA under the Data Use Agreement.

## Abbreviations

SPS: Subthreshold psychiatric symptoms
DSM-5: Diagnostic and Statistical Manual of Mental Disorders, 5th Edition
AUD: Alcohol use disorders
SUD: Substance use disorders
NESARC-III: The National Epidemiologic Survey on Alcohol and Related Conditions-III
NIAAA: National Institute on Alcohol Abuse and Alcoholism
AUDADIS-5: Alcohol Use Disorder and Associated Disabilities Interview Schedule
SE: Standard error
AOR: Adjusted odds ratios
CI: Confidence intervals
